# Editorial: Implications of gut-brain interactions for the effects of compounds derived from *Cannabis* or modulating the endocannabinoid system in physiological and pathological processes

**DOI:** 10.3389/fncel.2022.1042821

**Published:** 2022-10-20

**Authors:** Francesca Borrelli, Giuseppe Esposito, Jean-Pierre Montmayeur, Cristoforo Silvestri

**Affiliations:** ^1^Department of Pharmacy, School of Medicine and Surgery, University of Naples Federico II, Naples, Italy; ^2^Department of Physiology and Pharmacology, Sapienza University of Rome, Rome, Italy; ^3^Neuroscience Institute, Georgia State University, Atlanta, GA, United States; ^4^Centre de Recherche de l'Institut Universitarie de Cardiologie et de Pneumologie de Quebec (IUCPQ), Quebec, QC, Canada; ^5^Department of Medicine, Faculty of Medicine, Laval University, Quebec, QC, Canada

**Keywords:** gut-brain axis, endocannabinoid system, microbiota, cannabinoids, autism, cancer, colitis, opioid withdrawal

Over the past 35 years, considerable attention has been paid to the endocannabinoid system (ECS), which consists of endogenous cannabinoids, cannabinoid receptors, and the enzymes responsible for the synthesis and degradation of endocannabinoids. The ECS has been shown to play myriad physiological and pathological roles, including regulation of gut and brain homeostasis (Sharkey and Wiley, 2016). In addition to its endogenous functions, the ECS has been identified as a target of several exogenous molecules, including *Cannabis*-derived compounds that can directly activate the cannabinoid receptors or indirectly modulate the ECS by acting on the enzymes responsible for endocannabinoid metabolism (Martínez et al., 2020). With the discovery of the bidirectional interaction between the brain and gut (termed the gut-brain axis) and the influence of the microbiota on this axis, great efforts are being made to identify a role for the endocannabinoid system in these interactions. Indeed, increasing evidence supports the notion that the gut microbiome interacts with the ECS and that the latter plays a significant role in mediating enteric microbiota-host interactions (Lian et al., 2022). This special issue highlights published data from the literature and recent advances in the study of the interactions of the ECS system, the gut-brain axis, and the microbiota. The breadth of the topics explored speaks to the broad applicability of these interactions to host physiology and pathology. The collection includes three review articles focusing primarily on the role of the ECS and microbiota on the gut-brain axis, as well as two research articles that provide insights into the role of the ECS on gut and brain diseases. A review by Srivastava et al. describes in great detail the interaction between microbiota, intestinal endocannabinoid system, metabolism, and stress responses highlighting its central role in stress-induced changes in the gut-brain axis in relation to metabolic and mental health. In considering that, the authors emphasize the benefits of drugs targeting the microbiota and ECS to alleviate metabolic and stress-related disorders. Similarly, the review article by Coccurello et al. details the relationship between autism spectrum disorders (ASD), the microbiota, and the brain and gut ECS. It was highlighted that these systems are modifiable by, and integrate the response to, several external influences that are known to increase the probability of developing ASD. For instance, gut dysbiosis induced by antibiotic use and the absence of germs (germ-free mice) underlie changes in the ECS in the brain and gut associated with mood and neurodevelopmental disorders (NDD), thus suggesting that the ECS mediates many functions of bidirectional communication between the microbiota, gut, and brain. This hypothesis is further supported by the use of probiotics, which were able to reverse the ECS changes as well as the behavioral disturbances. It has also been demonstrated that gut dysbiosis and alteration of ECS signaling are involved in the development of ASD. However, it is still unclear if the disruption of the microbial ecosystem could be considered the primary event causing the disruption of ECS signaling or, conversely, whether the imbalance of the ECS system could trigger the disruption of gut microbial diversity described in ASD patients. A third review by Zaiachuk et al. summarizes preclinical studies supporting the potential use of synthetic and *Cannabis*-derived cannabinoids in the treatment of colorectal cancer (CRC). Specifically, the ECS has been reported to be altered in CRC and cannabinoids have been reported to target key signaling cascades involved in cancer development, including pathways affected by immunotherapy. Based on these effects, cannabinoids have been suggested to be used as adjunctive therapy for CRC. The information from the three reviews was supplemented by two research papers.

The research paper by Tartakover Matalon et al. provides new insights into the health potential of *Cannabis* in the treatment of ulcerative colitis. The authors demonstrate that *Cannabis* use is able to mitigate reductions in the levels of specific ECS lipids and that various measures of disease symptoms (bowel movements and general quality of life scores) corelated with ECS lipid levels in UC patients, which may explain the beneficial effects on disease symptoms in these patients. In addition, the authors point out the need for whole biopsy culture or 3D organoids to better recapitulate *in vivo* conditions. This work not only opens new perspectives on the impact of *Cannabis* on UC treatment but also suggests tools to study the underlying mechanisms.

Finally, the paper by Ayoub et al., demonstrates that opiate withdrawal is accompanied by changes in the levels of brain ECS-related oleoyl-amino acid congeners in association with changes in colonic microbiome community architecture due to significant changes in specific taxa. Furthermore, administration of the same oleoyl-amino acid congeners mitigated various symptoms in different opioid withdrawal models, though the effects were not always identical. Interestingly, colonic levels of different endocannabinoids and related *N*-acyl-serotonins were also found to be modified during opioid withdrawal, suggesting that these molecules, who's levels are also altered by microbiome disruption with antibiotics (Guida et al., 2018) may play a role in regulating gut-brain axis activity during opioid withdrawal.

Together, this collection of articles sheds light on the importance of the role of the ECS for the gut-brain axis in regulating diverse physiological and pathological processes such as autism spectrum disorders, ulcerative colitis, colorectal cancer immunotherapy and opioid withdrawal. Not only do they highlight the therapeutic potential of phytocannabinoids, endocannabinoids, and related bioactive lipid mediators in the treatment of pathologies of either the gut or the brain as well as those that are sensitive to alterations in the gut-brain axis, they also point out the promising role of microbiota, and personalization in this context.

## Author contributions

FB prepared the original draft. CS, GE, and J-PM critically reviewed and edited the manuscript. All authors contributed to the article and approved the submitted version.

## Conflict of interest

FB received research grants from GW pharmaceuticals to perform preclinical studies on phytocannabinoids and intestinal diseases. She holds patents on phytocannabinoids, colorectal cancer and inflammatory bowel diseases. The remaining authors declare that the research was conducted in the absence of any commercial or financial relationships that could be construed as a potential conflict of interest.

## Publisher's note

All claims expressed in this article are solely those of the authors and do not necessarily represent those of their affiliated organizations, or those of the publisher, the editors and the reviewers. Any product that may be evaluated in this article, or claim that may be made by its manufacturer, is not guaranteed or endorsed by the publisher.
